# Blood metabolomics reveals the therapeutic effect of *Pueraria* polysaccharide on calf diarrhea

**DOI:** 10.1186/s12917-023-03662-9

**Published:** 2023-07-29

**Authors:** Liuhong Shen, Yu Shen, Liuchao You, Yue Zhang, Zhetong Su, Guangneng Peng, Jun-liang Deng, Zhijun Zhong, Shumin Yu, Xiaolan Zong, Xiaofeng Wu, Yingkun Zhu, Suizhong Cao

**Affiliations:** 1 The Key Laboratory of Animal Disease and Human Health of Sichuan Province, The Medical Research Center for Cow Disease, College of Veterinary Medicine, Sichuan Agricultural University, Chengdu, 611130 Sichuan China; 2 Guangxi Innovates Medical Technology Co., Ltd. Lipu, Guangxi, 546600 China; 3 School of Agriculture & Food Science, University College Dublin, Belfield, Dublin, D04 V1W8 Ireland

**Keywords:** *Pueraria* polysaccharide, Calf diarrhea, Therapeutic efficacy, Metabolomic

## Abstract

**Background:**

Neonatal calf diarrhea (NCD) is typically treated with antibiotics, while long-term application of antibiotics induces drug resistance and antibiotic residues, ultimately decreasing feed efficiency. *Pueraria* polysaccharide (PPL) is a versatile antimicrobial, immunomodulatory, and antioxidative compound. This study aimed to compare the therapeutic efficacy of different doses of PPL (0.2, 0.4, 0.8 g/kg body weight (BW)) and explore the effect of plasma metabolites in diarrheal calves by the best dose of PPL.

**Results:**

PPL could effectively improve the daily weight gain, fecal score, and dehydration score, and the dosage of 0.4 g/kg BW could reach curative efficacy against calf diarrhea (with effective rates 100.00%). Metabolomic analysis suggested that diarrhea mainly affect the levels of taurocholate, DL-lactate, LysoPCs, and intestinal flora-related metabolites, trimethylamine N-oxide; however, PPL improved liver function and intestinal barrier integrity by modulating the levels of DL-lactate, LysoPC (18:0/0:0) and bilirubin, which eventually attenuated neonatal calf diarrhea. It also suggested that the therapeutic effect of PPL is related to those differential metabolites in diarrheal calves.

**Conclusions:**

The results showed that 0.4 g/kg BW PPL could restore the clinical score of diarrhea calves by improving the blood indexes, biochemical indexes, and blood metabolites. And it is a potential medicine for the treatment of calf diarrhea.

**Supplementary Information:**

The online version contains supplementary material available at 10.1186/s12917-023-03662-9.

## Background

Neonatal calf diarrhea (NCD) is a common disease of calves within four weeks old; the morbidity and mortality were about 55% and 15%, respectively [1]. NCD usually was caused by the interaction of environments (poor hygiene and high density), infectious factors (viruses, bacteria, parasites) [2], or non-infectious factors (stress, poor management) [3]. NCD is non-seasonal and prevalent worldwide, with primary symptoms including anorexia, elevated body temperature, depression, and diarrhea [4], affecting pastures' economic benefits. Antibiotics are often used to treat or prevent diarrhea in ruminants that are known to cause antibiotic-resistant bacteria [5]. Therefore, it is necessary to exploit natural agents for NCD management.

Bioactive compounds in plant extracts performed increasing importance in medicine development [6]. *Pueraria lobata*, which spreads widely in China, Japan, and Korea, has been used for thousands of years as medicine, fodder, and food. Polysaccharides and flavonoids are the main bioactive components of *Pueraria lobata* [7]. Polysaccharides like chitosan have been proven can effectively treat diarrhea [8]; furthermore, *Panax ginseng* polysaccharides [9] and red algae polysaccharides [10] have been used to alleviate diarrhea in various animal models. *Pueraria* polysaccharide (PPL), a compound of *Pueraria lobata,* has versatile bioactivities like antimicrobial [11], immunomodulatory [12], and antioxidant [13], and it can recover the expression and structure of tight junction protein of intestine, alleviating dextran sodium sulfate-induced colitis in mice [14]. It can also increase the beneficial bacteria and improve intestinal flora structure in antibiotic-induced diarrheal mice [15]. However, there is a lack of research focused on the therapeutic efficiency of PPL on newborn calf diarrhea.

This study investigated the therapeutic effect of PPL against calf diarrhea and determined the optimal dosage. Besides, very few studies have explored metabolomic profiling in the PPL treatment of neonatal calf diarrhea. In this study, we conducted comprehensive metabolomics profiling of plasma from diarrheal calves before and after treatment with PPL using liquid chromatography (LC)-TOF–MS.

## Materials and methods

### Preparation of PPL

PPL with 50.00% purity and average molecular weight (Mw) of 1.09 × 10^5^ Da [16], supplied by Guangxi Innovate Pharmaceutical Co., Ltd.

### Animals

The study was conducted on an intensively managed dairy farm in Sichuan, China. Dairy calves were offered 4 L colostrum within two h after birth, then housed individually with bedding material to avoid physical contact with each other. After the first day of life, the calves were fed milk from a bucket twice a day at 8:00 and 16:00, with free access to concentrate and water for the next seven days.

Thirty-six naturally infected Chinese Holstein dairy calves with typical diarrheal symptoms, and 12 healthy calves were selected from a unified pasture in Sichuan, China (calves with 5–7-day ages and 45–55 kg weights). The enrollment criteria for diarrheal calves were as follows: (1) calves with fecal scores > 2, (2) calves aged 1–30 days [17]. The calves enrolled in this study did not receive any other medications during the treatment with PPL. After the experiment, the animals returned to the herd for feeding.

### Drug administration

The day of enrollment was defined as D0. Thirty-six diarrheal calves were randomly divided into 3 groups (*n* = 12). The dose of each group was 0.2 g/kg, 0.4 g/kg, and 0.8 g/kg BW (dissolved in 100 mL 37℃ water) as low dose group, medium-dose group, and high-dose group, respectively. Administrate *Pueraria* polysaccharide Q.D P.O for five consecutive days. NCD was considered to end when their fecal score was ≤ 1 for two consecutive days. The whole experimental period was seven days.

### Therapeutic efficacies observation

During the experimental period (D0-D7), weighed calves before administration and calculated the daily weight gains, observed and recorded the mental status [18], dehydration status [19], and fecal status [17] (the criteria of these status scores were present in Table 1), heart ratio, respiratory ratio, and body temperature of calves, during this period, calculated the therapeutic effects and ascertain the optimal dosage.

**Table 1:** The scoring criteria of clinical examination

Items	Scoring Basis	Scores
Fecal status	Feces with normal shape and consistency	0
	Semi-formed or pasty feces	1
	Feces are not formed but can adhere to ground or bedding	2
	Watery feces	3
Mental status	Normal, respond quickly to external stimuli and vigorously	0
	Mild depressive retains sucking reflex without vitality	1
	Moderate depressive, stand after stimulation with a weak or irregular sucking reflex	2
	Severe depressive, unable to stand or without sucking reflex	3
Dehydration status	The hydration state is normal, tent test time of upper eyelid skin < 2 s	0
	Eyeball slightly sunken, tent test time of upper eyelid skin is > 2 s but < 4 s	1
	Sunken eyeball, dry nose, tent test time of upper eyelid skin > 5 s	2
	Severe sunken eyeball, cold ears, limbs and mouth, dry nose	3

### Blood collection for complete blood count

At the same time, the venous blood of the optimal dose group of PPL and control group were selected on D0, D3, D5, and D7 for complete blood count (CBC). Blood samples were collected via the jugular vein using the EDTA anticoagulation tube.

### Serum collection for biochemical indexes detection

Similarly, selected the venous blood of the optimal dose group of PPL and control group on D0, D3, D5, and D7 and centrifuged at 3000 r/min for 5 min to obtain the serum and stored at -20℃ for further biochemical indexes detection. Blood samples were collected via the jugular vein using the non-anticoagulation tube.

### Preparation of plasma samples for metabolomic analysis

Blood samples of healthy calves in the control group on D5 were collected and centrifuged at 3000r/min for 5 min to obtain the plasma and noted as group HS. Besides, the plasma collected from calves in optimal dosage groups on D0 and D7 was noted as group DS and TS, respectively. Blood samples were collected via the jugular vein using the EDTA anticoagulation tube. All the plasma was stored at -80℃ for further metabolomic analysis and comparison. The 3 sets of samples were mixed in equal amounts to prepare quality control (QC) samples, and 7 replicates were set up to evaluate system stability over the entire experiment before testing. The samples were sent to Shanghai Applied Protein Technology Co., Ltd. (Shanghai, China) for liquid chromatography-tandem mass spectrometry (MS/MS) analysis.

### Metabolomics analysis of blood samples

The samples were separated by UHPLC (1290 infinite LC, Agilent Technologies) HILIC column with 25℃ column temperature and 0.3 mL/min velocity. The mobile phase was A: 25 mM ammonium acetate and 25 mM ammonia in water, B: acetonitrile. The gradient was 85% B for 1 min, linearly reduced to 65% in 11 min, then reduced to 40% in 0.1 min and kept for 4 min, and then increased to 85% in 0.1 min, with a 5 min re-equilibration period employed.

After detection, the AB triple TOF 6600 mass spectrometer was used to obtain the samples' primary and secondary spectra. The ESI source conditions after HILIC chromatographic separation were as follows, Ion Source Gas1: 60, Ion Source Gas2: 60, Curtain gas: 30, source temperature: 600 ℃, Ion Spray Voltage Floating ± 5500 V, TOM MS scan m/z range: 60–1000 Da, product ion scan m/z range: 25–1000 Da, TOF MS scan accumulation time 0.20 s/spectra, product ion scan accumulation time: 0.05 s/spectra, the secondary mass spectrum was obtained by information-dependent acquisition (IDA) with high sensitivity mode selected, declustering potential (DP): ± 60 V, collision energy was fixed at 35 V ± 15 eV, IDA was set as follows: exclude isotopes within 4 Da, Candidate ions to monitor per cycle: 10.

### Data processing and statistical analysis

The raw data were converted into mzXML format by Proteo Wizard [20], and then the XCMS program was used for peak alignment, retention time correction, and peak area extraction [21]. For the data extracted using XCMS, ion peak data for which > 50% of the missing data within a group were deleted. After the data had been pre-processed by pareto-scaling, pattern recognition was performed using SIMCA-P software (version 14.1, Umetrics, Umea, Sweden), consisting of supervised orthogonal partial least squares discriminant analysis (OPLS-DA). The variable importance in the projection (VIP) value of each variable in the OPLS-DA model was calculated to indicate its contribution to the classification. Metabolites with the VIP value > 1 were further applied to Student’s *t*-test at the univariate level to measure the significance of each metabolite. The *P*-values less than 0.1 were considered statistically significant [22]. Compound identification of metabolites was performed by comparing accuracy m/z value (< 25 ppm) and MS/MS spectra with an in-house database established with available authentic standards. Random forest analysis, enrichment pathway analysis, and pathway impact analysis of metabolites that differed among groups were performed using MetaboAnalyst 5.0 (https://www.metaboanalyst.ca/) and Kyoto Encyclopedia of Genes and Genomes (KEGG, https://www.kegg.jp/kegg/).

Software of SPSS 19.0 was applied to check whether the data conform to the normal distribution, all measurement data were expressed by means ± SEM, one-way ANOVA analysis of Variance was used for comparison between groups, and *P* < 0.05 indicated a significant difference.

## Results

### The effects of Pueraria polysaccharide on the basic physiological indexes

As shown in Table 2, when diarrhea occurred, the daily gains of diarrheal calves decreased (*P* < 0.05), while after PPL treatment, the daily gains of all dose groups showed an increasing trend and showed no significant differences (*P* > 0.05) with control group except for low dose group on D7, suggesting a dose–effect relationship. As shown in Table S1, the body temperatures, respiratory rates, and heart rates of diarrheal calves were no different from the control group (*P* > 0.05), and the PPL treatment showed no significant effect on those indexes (*P* > 0.05).

**Table 2:** Effects of PPL on basic physiological indexes

Items	Treatment	Group				SEM
		Low dose	Medium dose	High dose	Control	
Daily gain	D1	0.33_e_^b^	0.32_d_^b^	0.32_e_^b^	0.84^a^	0.027
	D2	0.47_d_^b^	0.50_c_^b^	0.50_d_^b^	0.87^a^	0.031
	D3	0.51_d_^c^	0.63_b_^b^	0.64_c_^b^	0.86^a^	0.022
	D4	0.55_c,d_^c^	0.65_b_^b^	0.69_b,c_^b^	0.86^a^	0.019
	D5	0.62_b,c_^c^	0.71_a,b_^b^	0.70_b,c_^b^	0.82^a^	0.021
	D6	0.64_a,b_^c^	0.76_a_^b^	0.75_a,b_^b^	0.84^a^	0.024
	D7	0.71_a_^b^	0.79_a_^a,b^	0.80_a_^a,b^	0.85^a^	0.030
	*P* (dose)	< 0.001				
	*P* (time)	< 0.001				
	*P* (dose × time)	< 0.001				

For each indicator, different superscripted letters represent significant differences between different groups at the same time point (*P <* 0.05), while different subscripted letters represent significant differences with in one group between different time points (*P <* 0.05)

### The effects of Pueraria polysaccharide on the scores of clinical symptoms

As shown in Table 3, the fecal scores and dehydration scores of diarrheal calves were significantly higher than group C (*P* < 0.05), while after PPL treatment. The mental scores in the medium dose group on D0 were significantly higher than in other groups. After PPL treatment, mental and dehydration scores showed a decreasing trend and no significant differences with other groups on D7 (*P* > 0.05).

**Table 3:** Effects of PPL on scores of clinical symptoms

Items	Treatment	Group				SEM
		Low dose	Medium dose	High dose	Control	
Fecal scores	D0	2.17_a_^a^	2.33_a_^a^	2.08_a_^a^	0.00^b^	0.114
	D1	1.33_b,c_^a^	1.58_b_^a^	1.50_b_^a^	0.25^b^	0.165
	D2	1.42_b_^a^	1.25_b,c_^a^	1.08_b,c_^a^	0.08^b^	0.139
	D3	1.08_b,c_^a^	1.08_b_^a^	1.33_b_^a^	0.17^b^	0.169
	D4	1.17_b,c_^a^	1.00_b_^a^	1.00_b,c_^a^	0.17^b^	0.211
	D5	1.25_b,c_^a^	1.00_b_^a^	1.17_b_^a^	0.08^b^	0.209
	D6	0.75_b,c_^a^	0.42_c_^a,b^	0.58_c,d_^a^	0.00^b^	0.148
	D7	0.58_c_^a^	0.25_c_^a,b^	0.33_d_^a,b^	0.08^b^	0.165
	*P* (dose)	< 0.001				
	*P* (time)	< 0.001				
	*P* (dose × time)	< 0.001				
Dehydration scores	D0	1.17_a_^a^	1.25_a_^a^	1.25_a_^a^	0.00^b^	0.093
	D1	0.42_b_^a^	0.50_b_^a^	0.58_b_^a^	0.00^b^	0.112
	D2	0.25_b_	0.25_b,c_	0.17_c_	0.00	0.093
	D3	0.33_b_	0.08_c_	0.09_c_	0.00	0.089
	D4	0.30_b_	0.00_c_	0.00_c_	0.00	0.036
	D5	0.38_b_^a^	0.00_c_^b^	0.00_c_^b^	0.00^b^	0.058
	D6	0.25_b_	0.00_c_	0.00_c_	0.00	0.045
	D7	0.17_b_	0.00_c_	0.00_c_	0.00	0.042
	*P* (dose)	< 0.001				
	*P* (time)	< 0.001				
	*P* (dose × time)	< 0.001				
Mental scores	D0	0.25^a^	0.58_a_^b^	0.17^a^	0.00^a^	0.109
	D1	0.25	0.17_b_	0.25	0.00	0.093
	D2	0.08	0.17_b_	0.17	0.00	0.077
	D3	0.08	0.17_b_	0.25	0.00	0.082
	D4	0.33^a^	0.00_b_^b^	0.08^b^	0.00^b^	0.056
	D5	0.17	0.17_b_	0.25	0.00	0.089
	D6	0.08	0.08_b_	0.17	0.00	0.070
	D7	0.08	0.08_b_	0.17	0.00	0.070
	*P* (dose)	0.250				
	*P* (time)	0.0002				
	*P* (dose × time)	0.338				

For each indicator, different superscripted letters represent significant differences between different groups at the same time point (*P <* 0.05), while different subscripted letters represent significant differences with in one group between different time points (*P <* 0.05)

### The therapeutic effects of Pueraria polysaccharide on diarrheal calves

As shown in Table 4, the effective rate (The ratio of cured calves to the total number of diarrhea calves within seven days) of the medium dose group was the highest among the three treatment groups, thus ascertaining 0.4 g/kg BW (medium dose) as optimal dosage against calf diarrhea.

**Table 4:** The therapeutic effects of *Pueraria* polysaccharide on diarrheal calves

Items	Low dose group	Medium dose group	High dose group
Cured number within 7 days	8	12	11
Effective rate (%)	75.00%	100.00%	91.67%

### The effects of Pueraria polysaccharide on the CBC

**Fig. 1:**
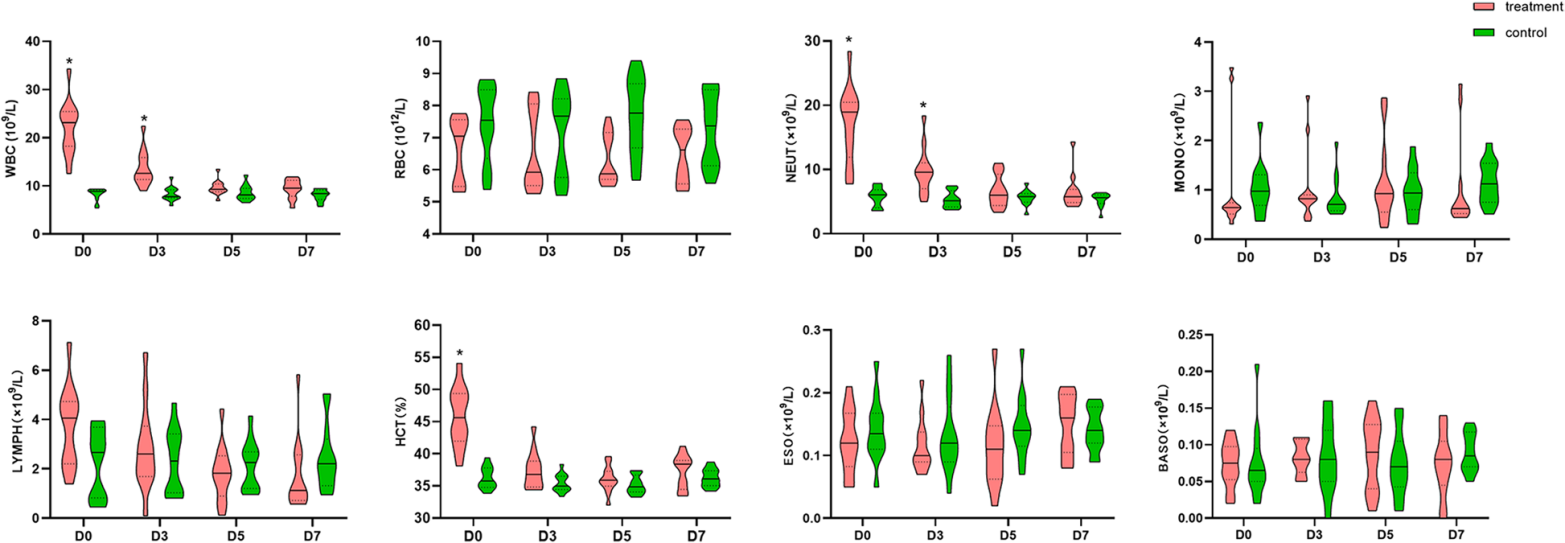
The effects of PPL on the CBC. Green box stands for control group; pink box stands for treatment group. D0 means the results of diarrheal calves before treatment with PPL. * *P* < 0.05, ** P < 0.01, ns = no significant

The effects of the optimal dose of PPL on the CBC of diarrheal calves, as shown in Fig. 1, the white blood cell (WBC), neutrophil count (NEUT), and hematocrit (HCT) of diarrheal calves were significantly higher than the control group on D0 and showed decreasing trends after treatment. There was no significant difference with the control group (*P* > 0.05). In contrast, there were no significant differences in red blood cell (RBC), lymphocyte counts (LYMPH), monocyte count (MONO), eosinophil (ESO), or basophil (BASO) with the control group (*P* > 0.05) on D0-D7.

### The effects of Pueraria polysaccharide on the biochemical blood indexes

**Fig. 2:**
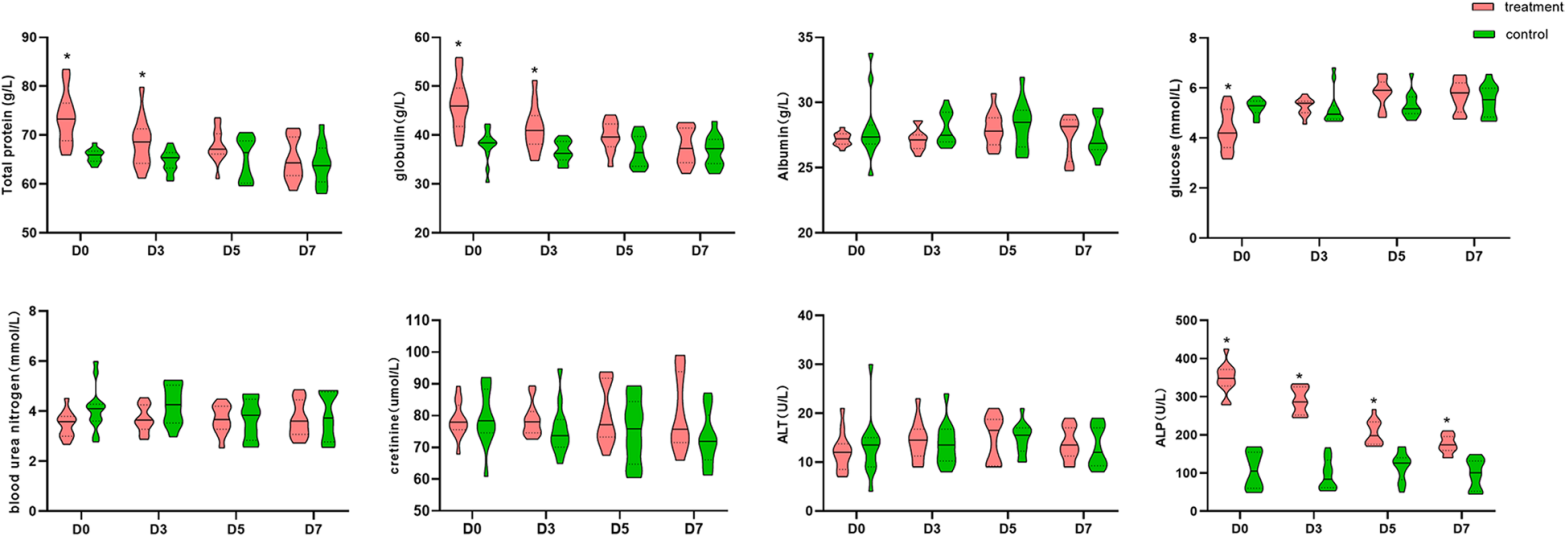
The effects of PPL on the serum biochemical indexes. Green box stands for control group; pink box stands for treatment group. D0 means the results of diarrheal calves before treatment with PPL. * *P* < 0.05, ** *P* < 0.01, ns = no significant

Similarly, we investigated the effects of the optimal dose of PPL on the biochemical blood indexes of diarrheal calves, and the results were present in Fig. 2. The levels of total protein (TP), globulin (GLOB), alkaline phosphatase (ALP) of diarrheal calves were significantly higher than the control group. The level of glucose (GLU) of diarrheal calves was significantly lower than the control group (*P* < 0.05), while all these indexes recovered to normal levels after treatment (*P* > 0.05), there were no significant differences of the albumin (ALB), blood urea nitrogen (BUN), creatinine (CREA), alanine aminotransferase (ALT) between medium-dose group and control groups.

### UHPLC-Q-TOF–MS method of validation

We compared the total ion chromatograms (TIC) of 7 samples in positive or negative ion modes, including the retention time, peak, intensity, and degree of separation. Overlap of the TIC of QC samples was good, indicating that the method used was robust, with high repeatability and stability. The sample TIC showed that the peak shape was intact and that adjacent peaks were well separated, indicating that the chromatographic and mass spectrometric conditions were suitable for sample identification (Fig S1). The smaller the relative standard deviation (RSD) of the ion peak abundance of QC samples is, the better the stability of the instrument is, which is an important index to reflect the quality of the data. In this experiment, the number of RSD ≤ 30% Peak in QC samples accounts for more than 80% of the total Peak number of QC samples, as shown in Fig S2, indicating that the stability of the instrument analysis system is good, and the data can be used for follow-up analysis.

### Identification of differential metabolites

**Fig. 3:**
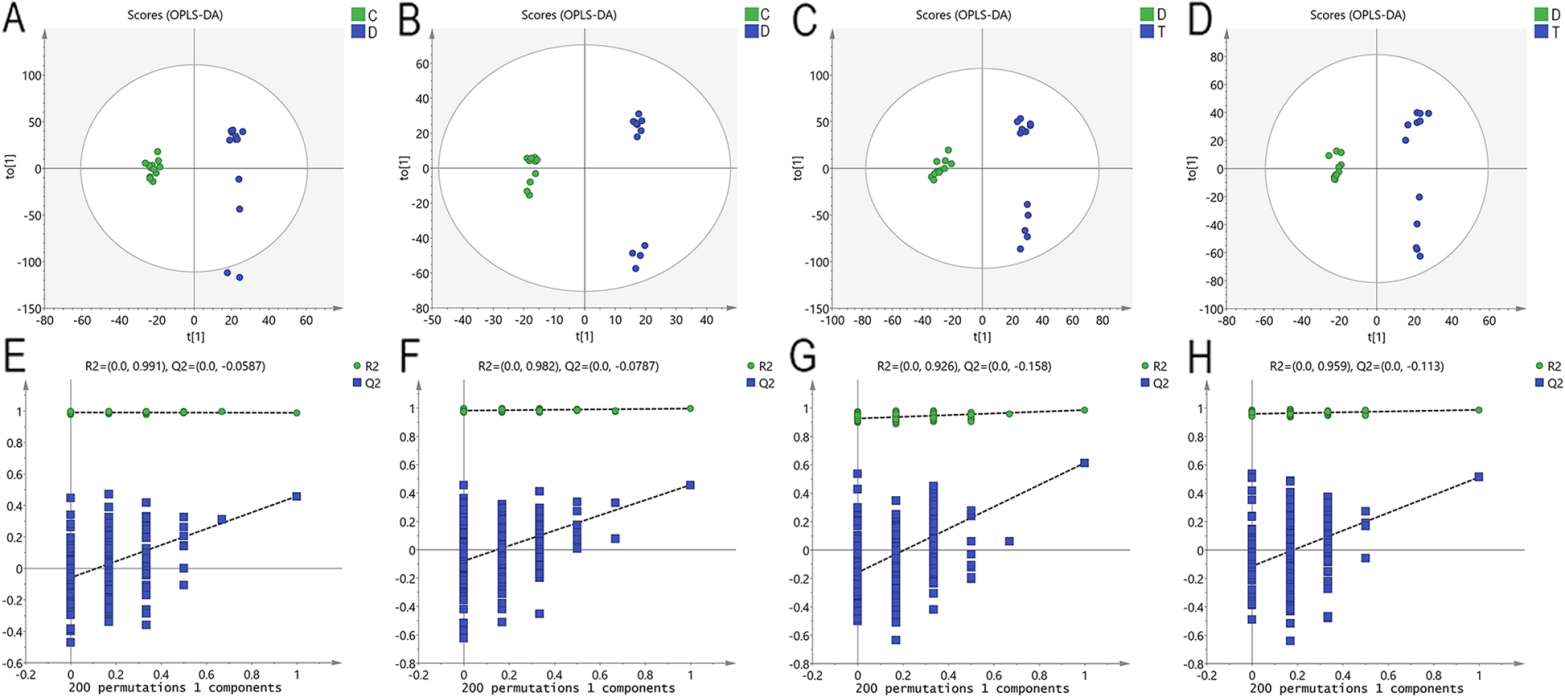
The scores plot of OPLS-DA and permutation tests. **A**, (**E**) and (**C**), (**G**) Orthogonal partial least square discriminant analysis (OPLS-DA) of scores and permutation test plots for the DS vs HS and TS vs DS samples analyzed in the positive ion mode, respectively. (**B**), **F** and (**D**), (**H**) Orthogonal partial least square discriminant analysis of scores and permutation test plots for the DS vs HS and TS vs DS samples analyzed in the negative ion mode, respectively. T [1] = first principal component. To [1]  = second orthogonal component. The intercept limit of Q^2^, calculated by regression line, is the plot of Q.^2^ from permutation test in the OPLS-DA model. HS = healthy calf plasma sample; DS = diarrheal calf plasma sample on D0; TS = PLP treatment diarrheal calf plasma sample on D5

**Fig. 4:**
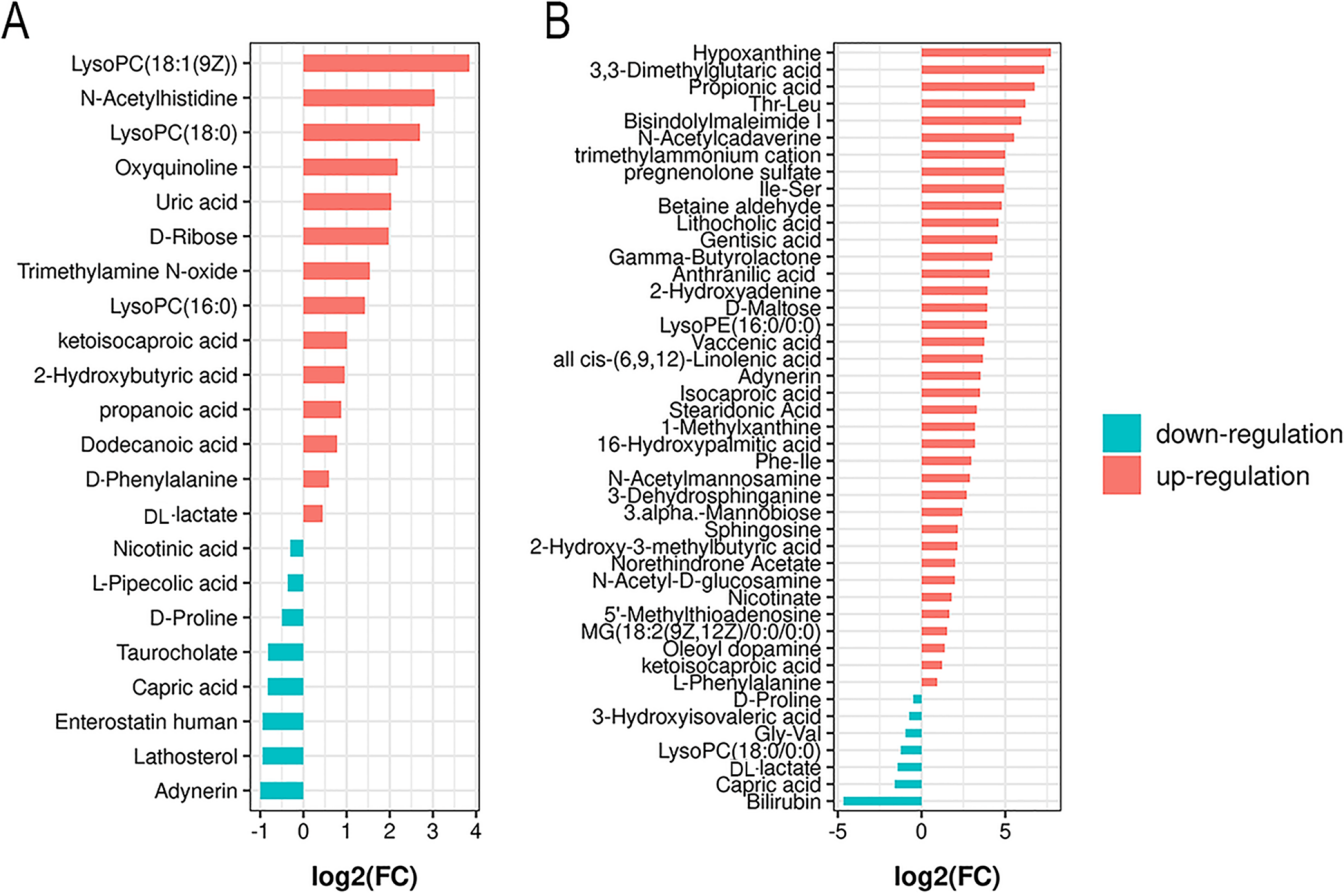
The FC barplot of differential metabolites in each group. **A**, **B** are the FC barplot of differential metabolites of DS vs HS, TS vs DS; red means up-regulation, blue means down-regulation

Potential biomarkers were analyzed using the multivariate analysis of OPLS-DA. Each point on the OPLS-DA score map represented a sample, and the position of each sample was determined by the type and content of its metabolites (Table S2). Compounds with a VIP > 1, fold change (FC) > 1, or < 1, and an independent t-test with *P* < 0.1 were initially screened as potential differential metabolites. The score plots of OPLS-DA and permutation tests shown in Fig. 3 indicated that the plasma metabolic profile of diarrheal calves was different from the control group, and there were also alterations of the metabolites profile in diarrheal calves after treatment with PPL. The intercepts of Q^2^ were < 0, indicating no overfittings of the model, and the differential metabolites can be identified according to it. There was a total of 22 differential metabolites identified between diarrheal calves and healthy calves (Table S3), with 14 metabolites found to increase and 8 observed to decrease. Similarly, 45 metabolites were found to significantly differ between PPL-treated calves and diarrheal calves (Table S4), with 38 metabolites found to increase and 7 observed to decrease. To compare the change of differential metabolites in each group, the differential plasma metabolites were shown by the FC barplot (Fig. 4).

### The change of key metabolites induced by PPL on diarrheal calves

**Fig. 5:**
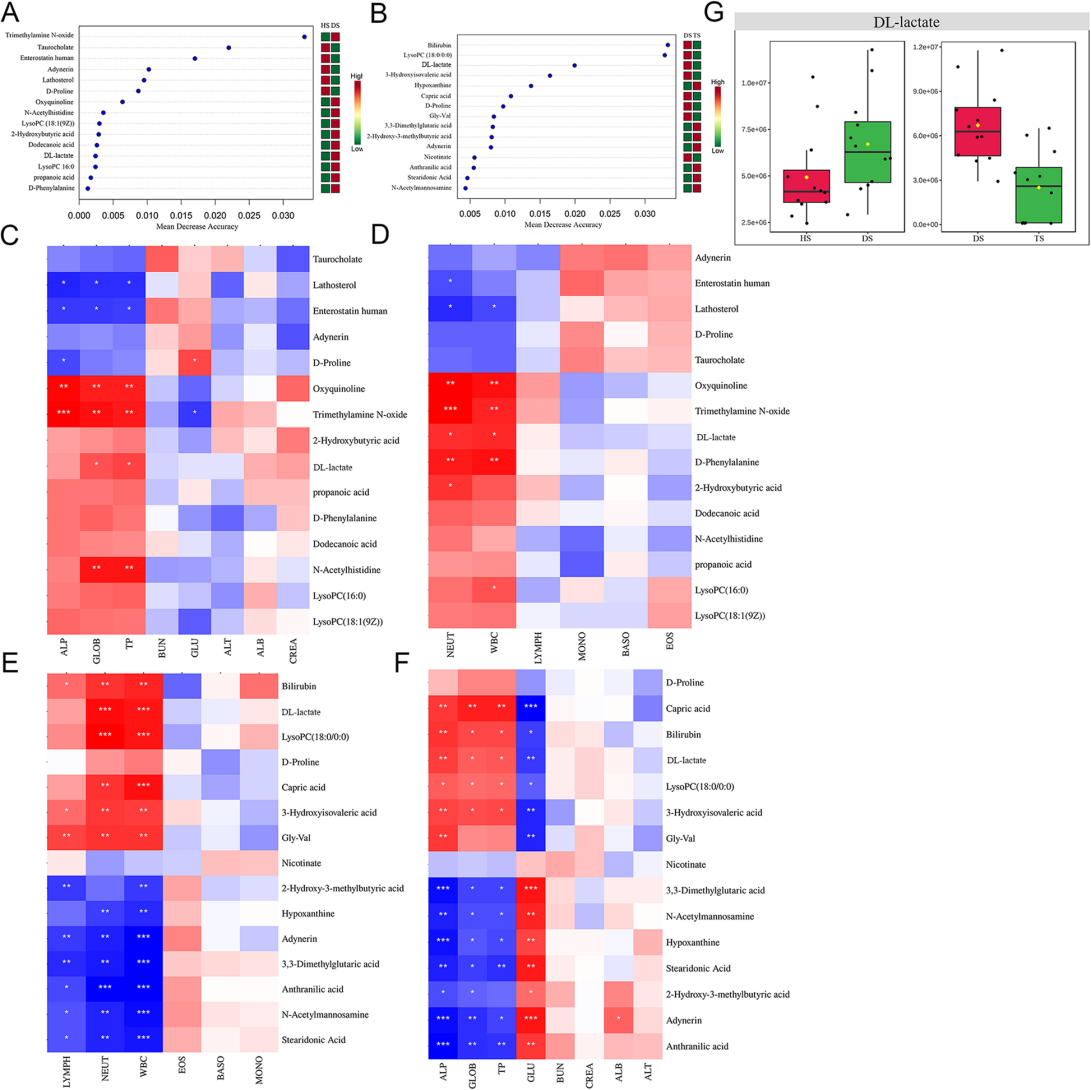
Potential information of differential metabolites. **A**, **B** the random forest supervised machine learning algorithm; C-F the correlation analysis of significantly altered metabolites with CBC and serum biochemistry indexes; G change of the DL-lactate concentration. ‘*’ indicates significant difference (*P* < 0.005). The red color and green color stand for different group, respectively

To further investigate the potential information of differential metabolites, the random forest supervised machine learning algorithm showed that 15 prominent metabolites contributed to the discrimination power of calf health status, including trimethylamine N-oxide (TMAO) and taurocholate; and 15 prominent metabolites related to the therapy of PPL, including bilirubin and LysoPC (18:0/0:0) (Fig. 5A, B). The relative rank of the relative abundance of metabolites biomarkers was plotted against the effect by the score of Mean Decrease Accuracy. Then the correlation analysis of significantly altered metabolites with CBC and serum biochemistry indexes was further revealed. As shown in Fig. 5, spearman rank correlation analysis indicated a strong positive correlation between TMAO and ALP (*r* = 0.64, *P* < 0.001), GLOB (*r* = 0.57, *P* < 0.01), TP (*r* = 0.55, *P* < 0.01), WBC (*r* = 0.52, *P* < 0.01) and NEUT (*r* = 0.65, *P* < 0.001) in the diarrheal calves. After being treated with PPL, bilirubin was positively correlated with ALP (*r* = 0.56, *P* < 0.01), WBC (*r* = 0.61, *P* < 0.01) and NEUT (*r* = 0.57, *P* < 0.01); similarly, LysoPC (18:0/0:0) was positively correlated with NEUT (*r* = 0.71, *P* < 0.001) and WBC (*r* = 0.64, *P* < 0.001). Notably, GLU was closely associated with DL-lactate (*r* = -0.54, *P* < 0.01) and stearidonic acid (*r* = 0.63, *P* < 0.01) after PPL treatment. It should be noted that the concentration of DL-lactate was altered in diarrheal calves before and after being treated with PPL(Fig. 5G). Our findings suggested that PPL administration could ameliorate diarrhea by modulating key plasma metabolites in diarrheal calves.

### Global metabolic pathways of metabolites

**Fig. 6:**
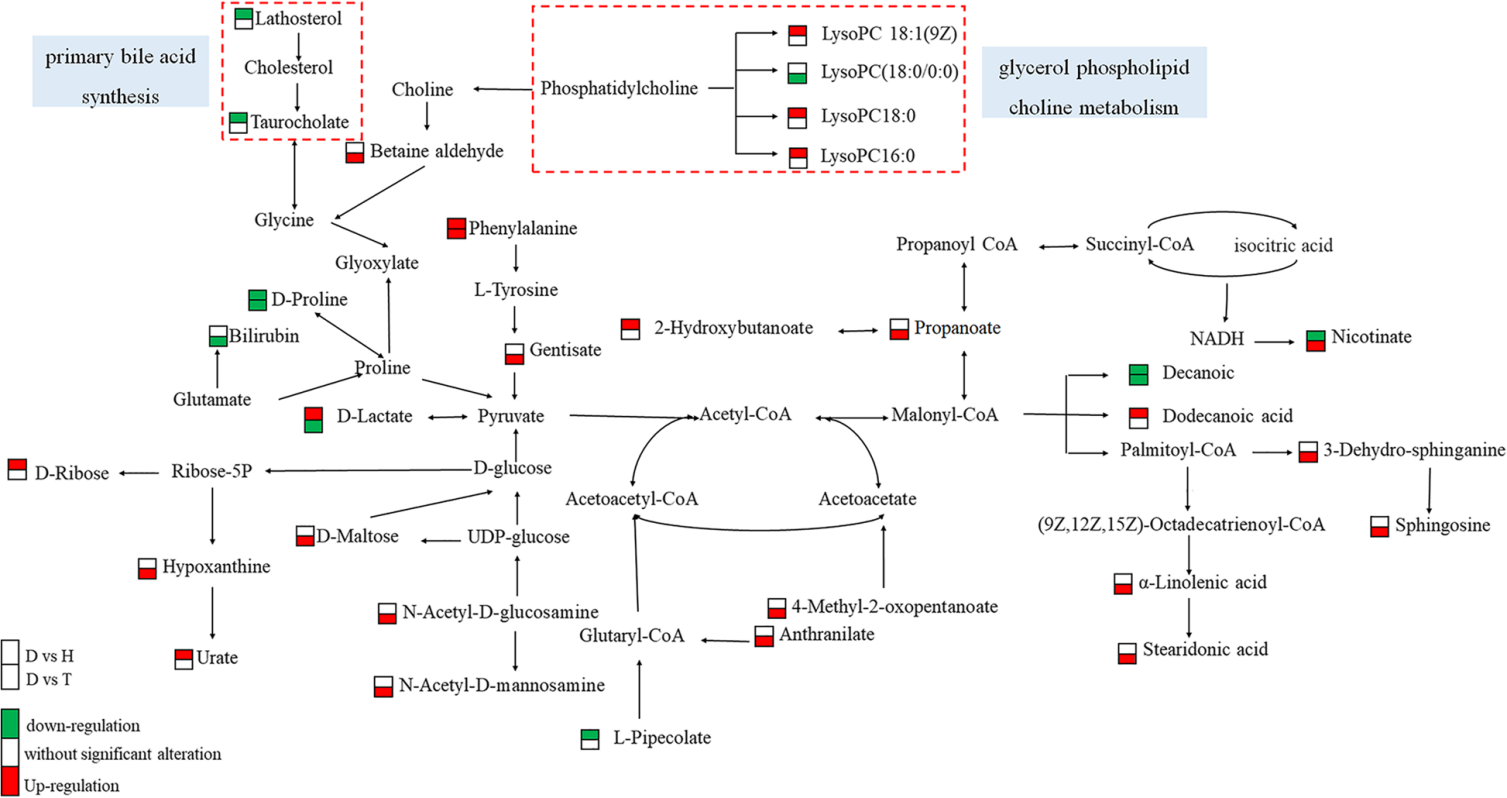
The global metabolic pathway of metabolites. The trisection rectangle represents the differential metabolites; the box on the top is DS vs HS group, and the box at the bottom is DS vs TS group; green represents downregulation, white represents no significant difference, red represents upregulation

Sketched the global metabolic pathways by the combination of KEGG pathway information. As shown in Fig. 6, the key metabolites mentioned above were mainly involved in 2 metabolic pathways, glycerol phospholipid choline metabolism and primary bile acid synthesis.

## Discussion

Apart from diluted soft faces, calf diarrhea also showed symptoms, including dehydration, weight loss, anorexia, and mental depression. Besides, diarrheal calves usually exhibited changes in complete blood count indexes and biochemical indexes [23]. In this study, PPL can alleviate the daily weight gain, fecal score, dehydration score, and mental score of diarrheal calves and reach curative efficacy (with a 75% cure rate). As signs of infection, the increased WBC and NEUT in diarrheal calves was coincided with another research about diarrheal calves [24], while after treatment of PPL, the WBC and NEUT returned and showed no significant difference with the control group (*P* > 0.05), indicating the amelioration of infectious and inflammatory degrees of diarrheal calves. Serum biochemical parameters are important indexes that reflect the health status of animals [25]. In the study, diarrheal calves showed increased serum ALP, globulin and decreased glucose. ALP was a marker of intestinal injury and increased obviously when intestinal damage [26]. After PPL administration, serum ALP showed a decreasing trend, indicating that PPL can promote intestinal integrity recovery. Globulin, an important component of the inflammatory microenvironment, is closely related to inflammatory status [27]. The recovery of globulin after PPL treatment also suggested the recovery of inflammatory and infectious status in diarrheal calves. After PPL treatment, serum glucose increased and exhibited no significant difference with the control group (*P* > 0.05) in diarrheal calves, demonstrating that PPL could recover the nutrition absorption and energy metabolism of diarrheal calves. Due to the limitation of the experimental conditions, the dehydration and ion disturbance of diarrhea calves were not analyzed in detail. Further study is needed.

As well known, diarrhea was an important factor leading to intestinal microbial imbalance [28]. Gut microbial dysbiosis can increase TMAO concentrations [29]. TMAO is the metabolite of choline and phosphatidylcholine from gut microbiota and hepatic flavin monooxygenases [30]. And in this study, the positive correlation between TMAO and ALP also indicated that diarrhea-induced intestinal damage in neonatal calves. We also found the level of taurocholate was decreased in diarrheal calves. The primary bile acid synthesis pathway, in which taurocholate takes part, is also downregulated. Bile acids secreted into the small intestinal are conjugated to taurine to form taurocholate [31]. Intestinal floras can modify the conjugated amino acids by secreting bile salt hydrolases [32]. Moreover, calves that suffer from diarrhea usually have weakened bile acid hepatointestinal circulation [33], which causes primary bile acid synthesis downregulation. Hence, we speculated that the down-regulation of taurocholate might relate to the increasing concentration of bile salt hydrolases, which reflected the intestinal flora disturbance, but more research was needed.

Usually, maintaining normal intestinal function architecture and functioning is essential for alleviating diarrhea. Our study noticed that PPL decreased the fecal score, which might be related to the DL-lactate change. The increases in the serum D-lactate have been reported to correlate with the extent of intestinal barrier dysfunction and diarrhea [34, 35]. D-lactate is produced only by colonic bacteria as a normal byproduct of bacterial fermentation. As the normal mucosal barrier is damaged and permeability increases, a large amount of D-lactate is released through the damaged intestinal mucosa into the peripheral blood, leading to increased blood levels of D-lactate. Because the liver cannot metabolize D-lactate, a rise in the serum concentration occurs [36]. Consistent with these findings, we noted a decrease in the serum DL-lactate contents following PPL treatment. However, it is a pity that our technology can only detect DL-lactate and cannot accurately distinguish its chiral structure, so subsequent experiments need specific detection of lactate to verify the experimental results.

Studies showed that diarrhea is usually followed by a negative energy balance [37]. Negative energy balance is characterized biochemically by the reduction in GLU concentrations [38]. Concurrently, we found DL-lactate was negatively correlated with GLU, then gradually recovered after PPL was treated. It may be due to the anorexia of diarrhea calves, resulting in poor nutrition absorption. However, PPL can improve the appetite of diarrhea calves by restoring their symptoms. The level of GLU was decreased in diarrheal calves. Increases in ALP and bilirubin levels were thought to be liver damage developed in calves with diarrhea [39]. Similarly, in this study, the levels of bilirubin and ALP were downregulated after PPL was treated. Therefore, we speculated that PPL could attenuate calf diarrhea by improving liver function.

In addition to damaging the intestinal integrity and liver function in neonatal calves, the induction of diarrhea triggers the inflammation response in the calf with diarrhea. LysoPC is generated by the enzyme phospholipase A2, which hydrolyses phosphatidylcholine at the sn-2 position [40], and participates in the component of biological membranes in animal cells [41]. The levels of the LysoPC family members, including LysoPC 16:0, LysoPC 18:0, and LysoPC 18:1(9Z), increased in diarrheal calves. LysoPCs were regarded as proinflammatory mediators, LysoPC 18:0 can induce the initiation of neutrophils [42], and stimulate the adhesion of eosinophils [43]. Diarrheal symptoms are usually related to systemic inflammatory responses, such as abnormal blood routine changes and biochemistry parameters [44], which could explain the increase of WBC and NEUT in diarrheal calves. Simultaneously, the down-regulation of LysoPC (18:0/0:0) also suggested that PPL attenuated system inflammatory response in diarrhea calves. We also found that the level of LysoPE (16:0) was increased in treated calves. The study indicated that LysoPE levels were reduced in the liver of mice with liver damage [45]. We speculated that the liver is a possible source of lipid perturbations in calves with diarrhea, which the following experiment could explore.

## Conclusion

The PPL showed a significant effect on calf diarrhea, and it can reach optimal therapeutic efficacy with a dose of 0.4 mg/kg/day. Diarrhea was associated with metabolic disorders in neonatal calves. Specifically, we found associations of diarrhea with shifts in taurocholate, DL-lactate, LysoPCs and intestinal flora-related metabolites, TMAO; while PPL improved liver function and intestinal barrier integrity by modulating the levels of DL-lactate, LysoPC (18:0/0:0) and bilirubin, which eventually ameliorated neonatal calf diarrhea. The only catch: the effects of PPL on the phenotype index of diarrheal calves, such as inflammatory factors, need further investigation.

## Supplementary Information

**Additional file 1: Table S1. **The scoring criteria of clinical examination.** Table S2.** Validation results of OPLS model.** Table S3.** Differential metabolites identified of C vs D groups in the positive or negative mode.** Table S4.** Differential metabolites identified of D vs T groups in the positive or negative mode.** Figure S1.** TIC of QC sample in positive and negative modes respectively.** Figure S2.** RSD of QC sample in positive and negative modes respectively.

## Data Availability

The datasets generated and/or analyzed during the current study are available from the corresponding author on reasonable request.

## Abbreviation

NCD: Neonatal calf diarrhea
PPL: Pueraria polysaccharide
CBC: Complete blood count
TOF/MS: Time-of-flight/ mass spectrometer
IDA: Information-dependent acquisition
OPLS-DA: Orthogonal partial least squares-discrimination analysis
VIP: Variable importance in the projection
WBC: White blood cell
NEUT: Neutrophil count
HCT: Hematocrit
RBC: Red blood cell
LYMPH: Lymphocyte counts
MONO: Monocyte count
ESO: Eosinophil
BASO: Basophil
TP: Total protein
GLOB: Globulin
ALP: Alkaline phosphatase
GLU: Glucose
ALB: Albumin
BUN: Blood urea nitrogen
CREA: Creatinine
ALT: Alanine aminotransferase
TIC: Total ion chromatograms
RSD: Standard deviation
FC: Fold change
TMAO: Trimethylamine N-oxide
