# Supplementary material for: Blood metabolomics reveals the therapeutic effect of Pueraria polysaccharide on calf diarrhea
Source: BMC Vet Res. 2023 Jul 29;19:98. doi: 10.1186/s12917-023-03662-9 (PMC10386334; doi:10.1186/s12917-023-03662-9)
Supplement: Supplementary file 1 — Additional file 1: Table S1. The scoring criteria of clinical examination. Table S2. Validation results of OPLS model. Table S3. Differential metabolites identified of C vs D groups in the positive or negative mode. Table S4. Differential metabolites identified of D vs T groups in the positive or negative mode. Figure S1. TIC of QC sample in positive and negative modes respectively. Figure S2. RSD of QC sample in positive and negative modes respectively. [file 12917_2023_3662_MOESM1_ESM.docx]

**Table S1** The scoring criteria of clinical examination

| Items | treatment | Group | | | | SEM |
| --- | --- | --- | --- | --- | --- | --- |
|  |  | low dose | medium dose | high dose | Control |  |
| Body temperature | D0 | 39.31 | 39.33 | 39.30 | 39.07 | 0.103 |
|  | D1 | 39.29 | 39.01 | 39.33 | 39.06 | 0.120 |
|  | D2 | 39.20 | 39.11 | 39.37 | 39.18 | 0.122 |
|  | D3 | 39.07 | 39.23 | 39.32 | 39.12 | 0.126 |
|  | D4 | 39.30 | 39.12 | 39.24 | 39.05 | 0.136 |
|  | D5 | 39.04 | 39.00 | 39.29 | 39.07 | 0.107 |
|  | D6 | 39.13 | 39.03 | 39.23 | 39.13 | 0.111 |
|  | D7 | 39.22 | 39.04 | 39.20 | 39.08 | 0.109 |
|  | *P* (dose) | 0.72 |  |  |  |  |
|  | *P* (time) | 0.01 |  |  |  |  |
|  | *P* (dose × time) | 0.98 |  |  |  |  |
| Respiratory rates | D0 | 52.50 | 51.00 | 57.17 | 53.08 | 4.247 |
|  | D1 | 51.17 | 50.17 | 61.00 | 52.58 | 4.730 |
|  | D2 | 51.67 | 51.33 | 58.67 | 56.67 | 3.721 |
|  | D3 | 50.33 | 54.33 | 58.92 | 52.50 | 3.214 |
|  | D4 | 49.92 | 50.67 | 59.17 | 54.50 | 3.551 |
|  | D5 | 51.33 | 52.33 | 59.25 | 55.33 | 3.267 |
|  | D6 | 50.25 | 51.33 | 58.75 | 56.67 | 2.995 |
|  | D7 | 50.83 | 49.92 | 58.50 | 52.58 | 3.506 |
|  | *P* (dose) | >0.999 |  |  |  |  |
|  | *P* (time) | <0.001 |  |  |  |  |
|  | *P* (dose × time) | >0.999 |  |  |  |  |
| Heart rates | D0 | 115.67 | 112.33 | 129.00 | 129.83 | 7.077 |
|  | D1 | 112.67 | 117.33 | 123.83 | 124.50 | 5.397 |
|  | D2 | 116.58 | 120.25 | 125.58 | 127.67 | 4.918 |
|  | D3 | 121.67 | 113.83 | 118.33 | 123.50 | 4.907 |
|  | D4 | 114.50 | 121.17 | 119.00 | 125.08 | 4.797 |
|  | D5 | 113.17 | 122.33 | 126.17 | 125.83 | 5.020 |
|  | D6 | 119.25 | 117.75 | 126.42 | 126.25 | 5.038 |
|  | D7 | 119.25 | 117.75 | 126.42 | 126.25 | 4.648 |
|  | *P* (dose) | 0.98 |  |  |  |  |
|  | *P* (time) | *P*<0.001 |  |  |  |  |
|  | *P* (dose × time) | 0.99 |  |  |  |  |

**Table S2.** Validation results of OPLS model

| POS | | | | | | |
| --- | --- | --- | --- | --- | --- | --- |
| Type | A | N | R2X(cum) | R2Y(cum) | Q2(cum) | Title |
| OPLS-DA | 1+1 | 24 | 0.378 | 0.737 | 0.0817 | D vs H |
| OPLS-DA | 1+1 | 24 | 0.445 | 0.751 | 0.594 | T vs D |
| NEG | | | | | | |
| Type | A | N | R2X(cum) | R2Y(cum) | Q2(cum) | Title |
| OPLS-DA | 1+1 | 24 | 0.336 | 0.696 | 0.271 | D vs H |
| OPLS-DA | 1+1 | 24 | 0.434 | 0.761 | 0.274 | T vs D |

**Table S3.** Differential metabolites identified of C vs D groups in the positive or negative mode

| **No.** | **Identification** | ***m/z*** | **RT(s)** | **VIP** | **Fold change** | ***P*-value** | **adduct** |
| --- | --- | --- | --- | --- | --- | --- | --- |
| 1 | Trimethylamine N-oxide | 76.07532 | 336.168 | 2.8403932 | 2.9112856 | 0.0016474 | (M+H)+ |
| 2 | Lathosterol | 369.35017 | 32.63 | 4.035593 | 0.5178434 | 0.0090515 | (M+H-H2O)+ |
| 3 | Oxyquinoline | 146.05881 | 90.3515 | 1.4614393 | 4.543195 | 0.0149019 | (M+H)+ |
| 4 | Nicotinic acid | 124.03799 | 218.7695 | 1.3218528 | 0.8099178 | 0.024813 | (M+H)+ |
| 5 | L-Pipecolic acid | 171.11289 | 272.578 | 5.2963505 | 0.7757847 | 0.0309279 | (M+CH3CN+H)+ |
| 6 | LysoPC(18:1(9Z)) | 544.33502 | 45.065 | 2.0421337 | 14.374977 | 0.0349919 | (M+Na)+ |
| 7 | Taurocholate | 533.32222 | 199.1485 | 1.5689724 | 0.5666077 | 0.0361279 | (M+NH4)+ |
| 8 | LysoPC(16:0) | 496.3364 | 163.246 | 3.5642494 | 2.6867617 | 0.0401743 | (M+H)+ |
| 9 | D-Phenylalanine | 207.11274 | 234.939 | 1.8087315 | 1.5120559 | 0.0403778 | (M+CH3CN+H)+ |
| 10 | D-Proline | 116.06986 | 346.925 | 1.5593485 | 0.7069937 | 0.0635175 | (M+H)+ |
| 11 | LysoPC(18:0) | 546.34979 | 124.465 | 2.3548783 | 6.4876866 | 0.0939858 | (M+Na)+ |
| 12 | Enterostatin human | 495.27264 | 66.376 | 1.0770586 | 0.5190448 | 0.0056462 | (M-H)- |
| 13 | ketoisocaproic acid | 129.05525 | 40.613 | 3.232388 | 2.017413 | 0.0206101 | (M-H)- |
| 14 | Uric acid | 167.01948 | 332.1985 | 1.080468 | 4.0966706 | 0.0353537 | (M-H)- |
| 15 | Adynerin | 515.29958 | 36.332 | 2.772862 | 0.4990181 | 0.0417684 | (M-H)- |
| 16 | 2-Hydroxybutyric acid | 103.03939 | 212.392 | 1.1874257 | 1.9411683 | 0.0528173 | (M-H)- |
| 17 | N-Acetylhistidine | 196.07067 | 323.467 | 1.7973983 | 8.2421513 | 0.0533759 | (M-H)- |
| 18 | propanoic acid | 165.05418 | 146.4095 | 1.0653949 | 1.837247 | 0.0548175 | (M-H)- |
| 19 | Capric acid | 171.13821 | 56.584 | 1.910275 | 0.5649254 | 0.0674019 | (M-H)- |
| 20 | D-lactate | 89.02494 | 259.472 | 6.1840176 | 1.3624683 | 0.0957776 | (M-H)- |
| 21 | D-Ribose | 149.0443 | 330.149 | 1.1882919 | 3.9315647 | 0.097087 | (M-H)- |
| 22 | Dodecanoic acid | 199.16871 | 39.2135 | 1.6273923 | 1.719836 | 0.098168 | (M-H)- |

^1^The adduct column showed the positive or negative mode. *m/z* = mass-to-charge ratio; RT = retention time; VIP = variable importance in projection. The same below.

**Table S4.** Differential metabolites identified of D vs T groups in the positive or negative mode

| **No.** | **Identification** | ***m/z*** | **RT(s)** | **VIP** | **Fold change** | ***P*-value** | **adduct** |
| --- | --- | --- | --- | --- | --- | --- | --- |
| 1 | LysoPC(18:0) | 568.33524 | 190.6445 | 2.2209236 | 0.4251165 | 0.0012338 | (M-H+2Na)+ |
| 2 | Gly-Val | 175.10588 | 310.56 | 1.1135948 | 0.5150676 | 0.0015665 | (M+H)+ |
| 3 | Anthranilic acid | 138.05283 | 315.878 | 1.1864583 | 16.796642 | 0.0170275 | (M+H)+ |
| 4 | Bilirubin | 585.26588 | 71.892 | 4.7649305 | 0.0397166 | 0.0201832 | (M+H)+ |
| 5 | Stearidonic Acid | 277.21409 | 38.1865 | 1.4552011 | 9.8892809 | 0.0326308 | (M+H)+ |
| 6 | N-Acetylmannosamine | 204.0852 | 260.045 | 1.8334036 | 7.3927545 | 0.0339253 | (M+H-H2O)+ |
| 7 | L-Phenylalanine | 166.08493 | 279.0125 | 1.0340697 | 1.9146622 | 0.0354238 | (M+H)+ |
| 8 | Sphingosine | 300.28766 | 101.305 | 1.3472861 | 4.4761986 | 0.0444564 | (M+H)+ |
| 9 | LysoPE(16:0/0:0) | 454.28938 | 47.283 | 1.5190832 | 15.101168 | 0.0445475 | (M+H)+ |
| 10 | Vaccenic acid | 283.26134 | 38.857 | 2.4648383 | 13.482541 | 0.0483973 | (M+H)+ |
| 11 | 2-Hydroxyadenine | 152.05517 | 242.8925 | 1.2092629 | 15.486586 | 0.0498282 | (M+H)+ |
| 12 | Oleoyl dopamine | 417.33271 | 37.5455 | 1.2691813 | 2.6241872 | 0.0514275 | M+ |
| 13 | all cis-(6,9,12)-Linolenic acid | 279.22972 | 167.019 | 1.2436738 | 12.748142 | 0.052333 | (M+H)+ |
| 14 | Phe-Ile | 279.1688 | 172.74 | 1.5247805 | 7.8503136 | 0.052576 | (M+H)+ |
| 15 | 3-Dehydrosphinganine | 300.28747 | 37.656 | 1.6384447 | 6.4668523 | 0.052753 | (M+H)+ |
| 16 | 16-Hydroxypalmitic acid | 295.2248 | 77.499 | 1.1170666 | 9.156949 | 0.0537708 | (M+Na)+ |
| 17 | Hypoxanthine | 137.04504 | 182.603 | 5.3093623 | 212.69944 | 0.0566985 | (M+H)+ |
| 18 | 3.alpha.-Mannobiose | 325.11035 | 402.3035 | 1.1603198 | 5.4037522 | 0.0569054 | (M+H-H2O)+ |
| 19 | Gamma-Butyrolactone | 87.04312 | 394.991 | 1.5885968 | 18.955261 | 0.066289 | (M+H)+ |
| 20 | D-Proline | 116.06986 | 346.925 | 1.4515477 | 0.7062585 | 0.066732 | (M+H)+ |
| 21 | 5'-Methylthioadenosine | 298.09497 | 105.345 | 1.8932056 | 3.1623027 | 0.0708897 | (M+H)+ |
| 22 | Ile-Ser | 260.15849 | 294.7435 | 1.0054938 | 30.531256 | 0.070995 | (M+CH3CN+H)+ |
| 23 | MG(18:2(9Z,12Z)/0:0/0:0) | 355.28184 | 36.134 | 1.2742476 | 2.8688491 | 0.0777364 | (M+H)+ |
| 24 | trimethylammonium cation | 146.11692 | 395.456 | 12.904315 | 31.819819 | 0.0824958 | M+ |
| 25 | N-Acetylcadaverine | 145.13247 | 324.472 | 1.499271 | 46.324661 | 0.0836145 | (M+H)+ |
| 26 | Isocaproic acid | 158.1162 | 51.271 | 1.1510291 | 11.336238 | 0.0862821 | (M+CH3CN+H)+ |
| 27 | Nicotinate | 124.03799 | 218.7695 | 2.2772332 | 3.4696951 | 0.0913387 | (M+H)+ |
| 28 | N-Acetyl-D-glucosamine | 222.09557 | 263.0265 | 1.0443359 | 3.9968693 | 0.0936289 | (M+H)+ |
| 29 | Betaine aldehyde | 162.11123 | 313.853 | 2.0534069 | 27.870892 | 0.0944922 | (M+CH3COO+2H)+ |
| 30 | Thr-Leu | 233.14811 | 243.547 | 1.1608199 | 74.389912 | 0.0968537 | (M+H)+ |
| 31 | Adynerin | 515.29958 | 36.332 | 2.8218867 | 11.493232 | 1.385E-05 | (M-H)- |
| 32 | D-lactate | 89.02494 | 259.472 | 10.121349 | 0.3719989 | 0.0005001 | (M-H)- |
| 33 | Capric acid | 171.13821 | 56.584 | 1.8094146 | 0.328988 | 0.0013644 | (M-H)- |
| 34 | 3-Hydroxyisovaleric acid | 99.04476 | 376.547 | 1.2579348 | 0.5987435 | 0.0014019 | (M-H2O-H)- |
| 35 | Norethindrone Acetate | 339.19643 | 27.217 | 1.9340319 | 4.0320522 | 0.0069306 | (M-H)- |
| 36 | Bisindolylmaleimide I | 411.17958 | 30.8405 | 1.5852589 | 62.26702 | 0.0429255 | (M-H)- |
| 37 | D-Maltose | 323.09586 | 268.91 | 1.0433567 | 15.291748 | 0.0529552 | (M-H2O-H)- |
| 38 | 2-Hydroxy-3-methylbutyric acid | 117.05496 | 138.652 | 1.1876077 | 4.4399922 | 0.0640303 | (M-H)- |
| 39 | 3,3-Dimethylglutaric acid | 159.06647 | 262.23 | 1.7846042 | 161.77703 | 0.0649738 | (M-H)- |
| 40 | 1-Methylxanthine | 165.04072 | 116.1425 | 1.0764088 | 9.1890709 | 0.0747031 | (M-H)- |
| 41 | pregnenolone sulfate | 395.18541 | 29.457 | 2.5392083 | 30.974899 | 0.0751665 | (M-H)- |
| 42 | Lithocholic acid | 375.28975 | 62.607 | 1.9903777 | 24.154984 | 0.0770652 | (M-H)- |
| 43 | ketoisocaproic acid | 129.05525 | 40.613 | 2.9113305 | 2.3560207 | 0.0788831 | (M-H)- |
| 44 | Propionic acid | 73.03006 | 172.9515 | 3.8688953 | 108.12992 | 0.0810726 | (M-H)- |
| 45 | Gentisic acid | 153.01842 | 57.461 | 1.5519891 | 23.160887 | 0.0975259 | (M-H)- |


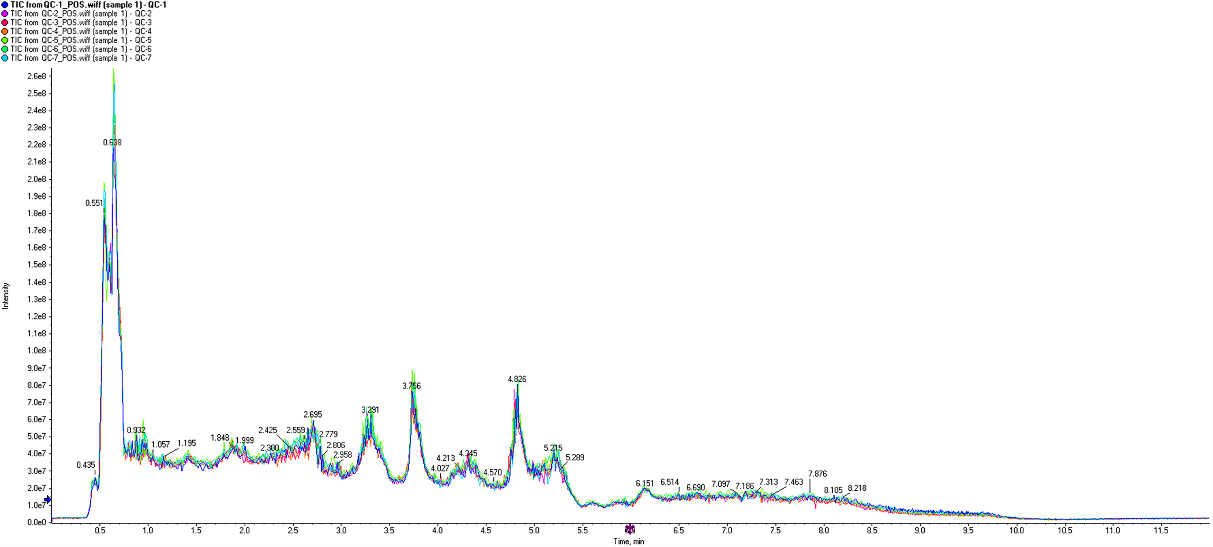


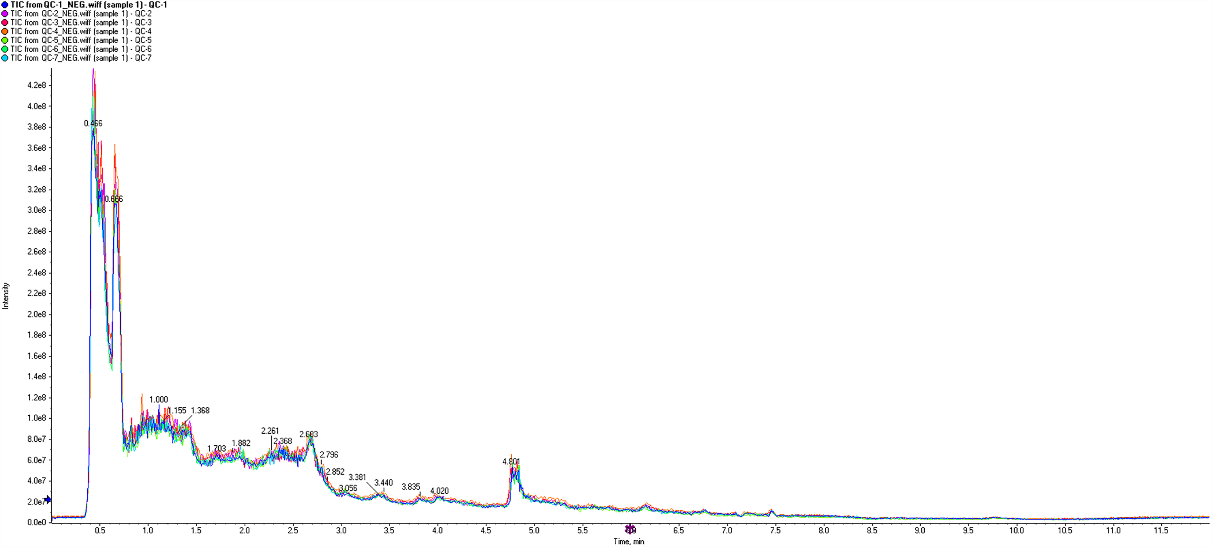


**Figure S1.** TIC of QC sample in positive and negative modes respectively.

| 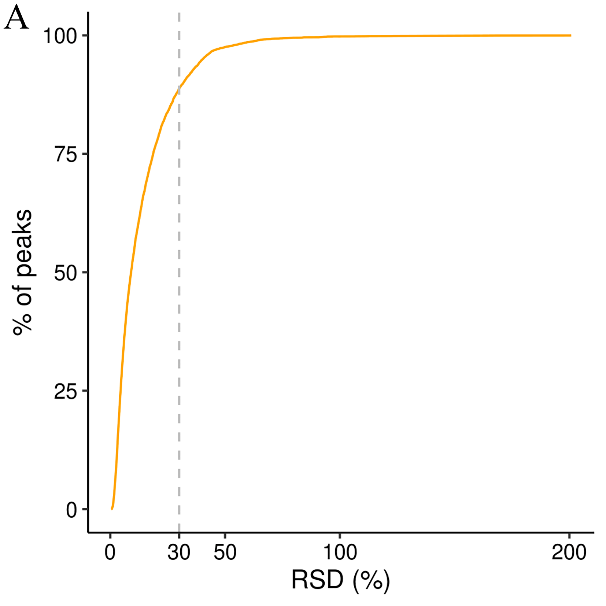 | 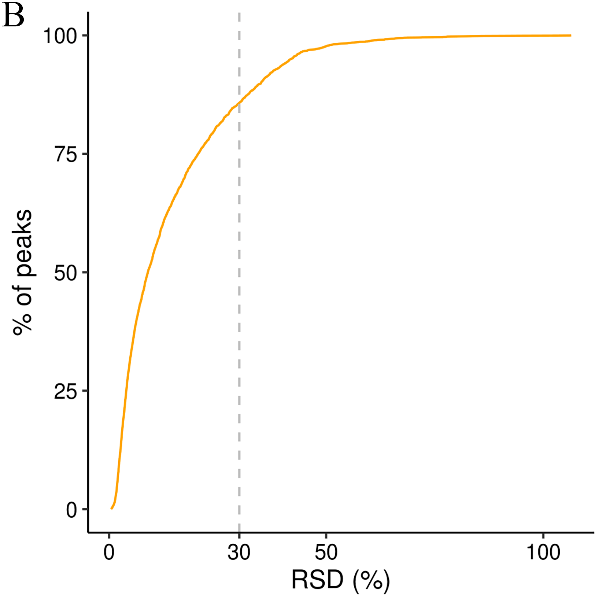 |
| --- | --- |
| **Figure S2.** RSD of QC sample in positive and negative modes respectively. | |
